# Microwave-Assisted Extraction of Polyphenols from *Camellia oleifera* Fruit Hull

**DOI:** 10.3390/molecules16064428

**Published:** 2011-05-27

**Authors:** Liangliang Zhang, Yongmei Wang, Dongmei Wu, Man Xu, Jiahong Chen

**Affiliations:** 1 Institute of Chemical Industry of Forest Products, CAF, Jiangsu Province, Nanjing 210042, China; 2 National Engineering Lab. for Biomass Chemical Utilization, Jiangsu Province, Nanjing 210042, China; 3 Key and Open Laboratory on Forest Chemical Engineering, SFA, Jiangsu Province, Nanjing 210042, China; 4 Key Lab. of Biomass Energy and Material, Jiangsu Province, Nanjing 210042, China; 5 Institute of New Technology of Forestry, CAF, Beijing 100091, China

**Keywords:** *Camellia oleifera*, polyphenols, microwave-assisted extraction, response surface methodology

## Abstract

The abundant fruit hulls of tea-oil tree (*Camellia oleifera*) are still underutilized and wastefully discaded to pollute the environment. In order to solve this problem and better utilize the fruit hulls of *C. oleifera*, a microwave-assisted extraction system was used to extract their polyphenols using water as the extraction solvent. A central composite design (CCD) was used to monitor the effects of three extraction processing parameters – liquid:solid ratio (mL/g), extraction time (min) and extraction temperature (°C) – on the polyphenol yield (%). The results showed that the optimal conditions were liquid:solid ratio of 15.33:1 (mL/g), extraction time of 35 min and extraction temperature of 76 °C. Validation tests indicated that under the optimized conditions the actual yield of polyphenols was 15.05 ± 0.04% with RSD = 0.21% (*n* = 5), which was in good agreement with the predicted yield. Phenolic compounds in the extracts were analysed by HPLC, and gallic acid was found to be the predominant constituent. The total flavonoid content in the extracts was determined and high total flavonoid content was revealed (140.06 mg/g dry material).

## 1. Introduction

Tea-oil tree (*Camellia oleifera*) is the most important edible oil tree growing specifically in China, and the oil from its seed is high-grade substance abundant in unsaturated fatty acids and vitamins. *C. oleifera* is mainly distributed in south China (3.5 million hm^2^) and has been developed into the main non-wood forest tree crop, which yields about 5.6 million tons of *C. oleifera* fruits each year [1]. The seed of *C. oleifera* has 55% tea-oil, whose oiliness has been proved to be far better than that of palm, rape, bean and peanut oil, and could even exceed that of olive oil, as tea-oil is abundant in unsaturated fatty acids (more than 90%) and vitamins [2]. The fruit hull, a by-product of obtaining tea-oil from *C. oleifera* seed, accounts for more than 60% weight of the single *C. oleifera* fruit, suggesting that the fruit hull constitutes a very abundant *C. oleifera*-derived resource. The hull contains many chemical components, including lignin, pentosan and phenolic compounds, which are raw materials for producing furfural, xylitol and tannic extract [3,4]; moreover, *C. oleifera* hulls can be used to produce activated carbon and protein forage [5]. However, because of the lack of research on high value-added utilization of *C. oleifera* fruit hulls, this abundant resource is still under-utilized and wastefully discarded. Therefore, the development of integrative utilization and high added-value products from the fruit hull of *C. oleifera* could benefit the rapid and sustainable development of the *C. oleifera* industry, and present an additional source of income for farmers in the Chinese countryside [3]. In order to solve this problem and better utilize the fruit hull of *C. oleifera*, microwave-assisted extraction (MAE) technology was used to extract the polyphenols from fruit hull of *C. oleifera* to optimize the extraction process.

Because of the inherent advantages in reduction of extraction time and solvent, MAE has attracted significant interest over the past decade. Conventional extraction methods are typically associated with high solvent consumption, longer extraction times and increased risk of degradation of thermo-labile constituents. In MAE, the solvent and sample are contained in sealed extraction vessels under controlled temperature and pressure conditions. The closed vessels allow the temperature of the solvent to rise well above its boiling point, which shortens extraction time and subsequently increases extraction efficiency.

In the past, the majority of work [6,7,8] on MAE has been focused on the extraction of organic compounds from soil and essential oils from plants; however, its efficacy in the extraction of phenolic compounds from plant material is only now being investigated [9,10,11]. Kerem *et al.* [12] compared the efficiency of Soxhlet extraction to MAE in the removal of saponins from chickpea, and it was found that extract profiles obtained after 20 min using the MAE process, were similar to those observed after 3 h of Soxhlet extraction. Li *et al.* [13,14] studied the effects of focused MAE on the extraction of phenolic acids (gallic, cholorgenic and caffeic acids) from *Eucommia ulmodies*, a plant widely used in Chinese medicine due to its antibacterial, antimutagenic and antioxidant properties. The best extraction conditions were found to be the use of 50% microwave power for 30 s, and a solvent volume to skin ratio of 10 mL/g.

The objectives of the current study were to investigate the effects of MAE on the extraction efficiency of polyphenols from *C. oleifera* fruit hulls and to optimize the extraction process using response surface methodology (RSM). RSM is a collection of statistical and mathematical techniques and is effective for responses that are influenced by many factors and their interactions [15]. To maximize levels of polyphenol yield, three extraction conditions – liquid:solid ratio (mL/g), microwave extraction time (min) and microwave extraction temperature (°C) – were optimized in this study. Up to now, it is the first description of an optimization of the process for extraction of polyphenols from fruit hulls of *C. oleifera* using RSM.

## 2. Results and Discussion

### 2.1. Extraction Model and Statistical Analysis

A total of 20 runs were used to optimize the three individual parameters in the CCD applied to the production of polyphenols from *C. oleifera* fruit hulls. The response values at different experimental combination for coded variables were listed in Table 1.

**Table 1:** The experimental design for response surface analysis in terms of coded values and the experimental results.

Experiment No.	Extraction temperature, *x*_1_ (°C)	Liquid:solid ratio, *x*_2_ (v/w)	Extraction time, *x*_3_ (min)	Response, *Y* (%)
1	−1	−1	−1	9.42
2	1	−1	−1	13.02
3	−1	−1	1	11.29
4	1	−1	1	12.39
5	−1	1	−1	12.04
6	1	1	−1	13.86
7	−1	1	1	13.21
8	1	1	1	13.06
9	−1.68	0	0	11.70
10	1.68	0	0	15.04
11	0	0	−1.68	12.58
12	0	0	1.68	12.68
13	0	−1.68	0	10.52
14	0	1.68	0	13.54
15	0	0	0	13.47
16	0	0	0	13.51
17	0	0	0	13.52
18	0	0	0	13.40
19	0	0	0	12.56
20	0	0	0	12.48

The polyphenol yield ranged from 9.42% to 15.04%. By applying multiple regression analysis on the experimental data, the response variable and the test variables were related by the following second-order polynomial Equation:

*Y* = 13.17 + 0.88*X*_1_ + 0.13*X*_2_ + 0.81*X*_3_ – 0.56*X*_1_*X*_2_ – 0.38*X*_1_*X*_3_ – 0.11*X*_2_*X*_3_ – 0.00046*X*_1_^2^ – 0.26*X*_2_^2^ – 0.48*X*_3_^2^ (1)

The significance of each coefficient was determined using the *F*-test and *p*-values (Table 2). The corresponding variables are more significant if the absolute *F*-value becomes greater and the *p*-value becomes smaller [16]. It can be seen that the variables with the largest effect were the linear terms of extraction time (*x*_1_), extraction temperature (*x*_3_) and the quadratic term of liquid:solid ratio (*x*_2_^2^), extraction temperature (*x*_3_^2^), followed by the interaction effects of extraction time and liquid:solid ratio (*x*_1_*x*_2_), extraction time and extraction temperature (*x*_1_*x*_3_). The results suggest that the change of extraction time and extraction temperature had highly significant effects on the yield of polyphenols (*p* < 0.0001) from the fruit hull of *C. oleifera*.

**Table 2:** Estimated regression model of relationship between response variables (polyphenol yield) and independent variables (*x*_1_, *x*_2_, *x*_3_).

Variables	DF	SS	MS	*F*-value	*p*-value
*x*_1_	1	10.5593	10.5593	53.6307	<0.0001
*x*_2_	1	0.2288	0.2288	1.1619	0.3064
*x*_3_	1	9.0555	9.0555	45.9932	<0.0001
*x*_1_*x*_2_	1	2.4921	2.4921	12.6576	0.0052
*x*_1_*x*_3_	1	1.1521	1.1521	5.8515	0.0361
*x*_2_*x*_3_	1	0.0955	0.0955	0.4849	0.5021
*x*_1_^2^	1	0.0000	0.0000	0.0000	0.9969
*x*_2_^2^	1	0.9907	0.9907	5.0319	0.0487
*x*_3_^2^	1	3.2544	3.2544	16.5292	0.0023

Analysis of variance (ANOVA) for the model is given in Table 3. The coefficient of determination (*R*^2^) of the predicted model was 0.9334, suggesting a good fit; the predicted model seemed to reasonably represent the observed values. Thus, we conclude that the response was sufficiently explained by the model.

**Table 3:** Variance analysis of the second-order regression model on polyphenol yield.

Source	DF	SS	MS	*F*-value	*p*-value
Model^a^	9	27.57	3.06	15.56	<0.0001
Linear	3	19.84	6.61	10.91	0.0004
Quadratic	3	3.99	1.33	6.76	0.0091
Cross-product	3	3.74	1.25	2.72	0.0874
Error	10	1.97	0.20		
Total	19	29.54	161.86		

^a^ The coefficient of determination (*R*^2^) of the predicted model was 0.9334.

### 2.2. Optimization of the Procedure by RSM

**Figure 1:**
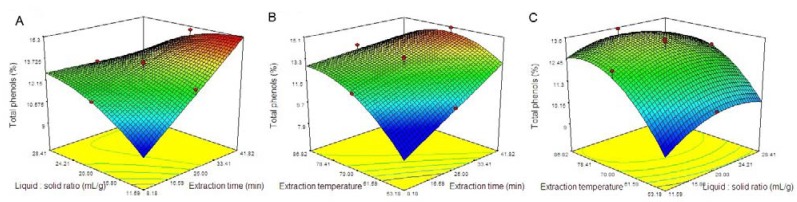
Response surface plots of the effect of (**A**) liquid:solid ratio and extraction time; (**B**) extraction temperature and extraction time; (**C**) extraction temperature and liquid:solid ratio on polyphenol yield.

Equation 1 allowed the prediction of the effects of the three factors on the polyphenol yield. Three independent response surface plots and their respective contour plots are shown in Figure 1. Two variables within the experimental rang were depicted in 3D surface plots while the other variable was kept constant at zero level. The shapes of the contour plots, circular or elliptical indicated whether the mutual interactions between the variables were significant or not [17,18].

As shown in Figure 1, the increased ratio of liquid:solid (*X*_2_) and extraction temperature (*X*_3_) up to a threashold level led to increased polyphenol yield (see Figure 1B and C). Beyond this level, the polyphenol yield slightly decreased, which indicated that a greater yield could be achieved if the moderate liquid:solid ratio (*X*_2_) and extraction temperature (*X*_3_) were selected. Meanwhile, extraction time (*X*_1_) had a positive impact on the polyphenol yield. There is an increase in the polyphenol yield with an increase in extraction time (see Figure 1A and B). Therefore, it could be concluded that the optimal conditions for microwave-assisted extraction of polyphenol yield from *C. oleifera* were a liquid:solid ratio of 15.33:1 (mL/g), microwave extraction time of 35 min, and microwave extraction temperature of 76 °C.

### 2.3. Validation of the Model

In order to validate the adequacy of the model equation (2), a verification experiment was carried out under the optimized conditions mentioned above. Under the optimal conditions, the model predicted a maximum response of 15.09%. A mean value of 15.05 ± 0.04% with RSD = 0.21% (*n* = 5), obtained from actual experiments, demonstrated the validation of the optimized extraction model. The good correlation between these results undoubtedly confirmed that the model was adequate for reflecting the predicted optimization.

### 2.4. HPLC Analysis Extracts

**Figure 2:**
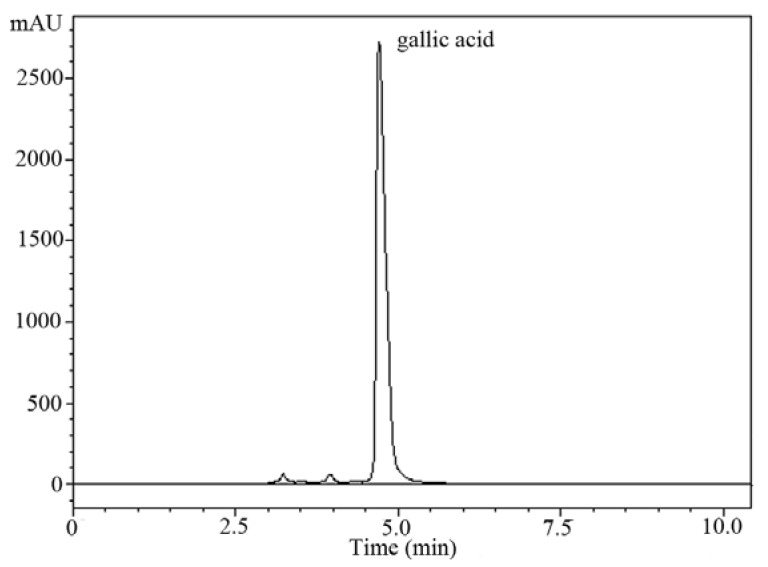
Concentrations of gallic acid recovered from *C. oleifera* fruit hull extracts.

RP-HPLC coupled with UV-Vis SPD detector was employed to separate, identify and quantify the phenolic compounds in the *C. oleifera* fruit hull extracts. The concentrations were determined by calculating the HPLC peak areas, which are proportional to the amount of analyte in a peak and presented as the mean of three determinations which were highly repeatable. Figure 2 shows the chromatogram of *C. oleifera* fruit hull extracts. The main phenolic compound in the extracts (66.18 mg/g dry material) was identified as gallic acid according to its retention time and spectral characteristics of their peak against the standard, as well as by spiking the samples with standards. 

### 2.5. Total Flavonoid Content

The total flavonoid content in the extracts of *C. oleifera* fruit hull was determined and high total flavonoid content was revealed (140.06 mg/g dry material).

## 3. Experimental

### 3.1. Plant Materials and Chemicals

The fruit hulls of *C. oleifera* were sampled from Bama, Guangxi Province (P.R. China), during October-November, 2009. The samples were shed-dried, pulverized and stored in airtight containers for further extraction. Gallic acid and quercetin were purchased from Sigma (St. Louis, MO, USA). Folin-Ciocalteu reagent was purchased from China Sinopharm Chemical Reagent Co., Ltd. Trifluoroacetic acid (TFA) was of analytical reagent (AR) purity grade. CH_3_CN used for the analysis was of HPLC grade. 

### 3.2. Extraction Procedure

**Figure 3:**
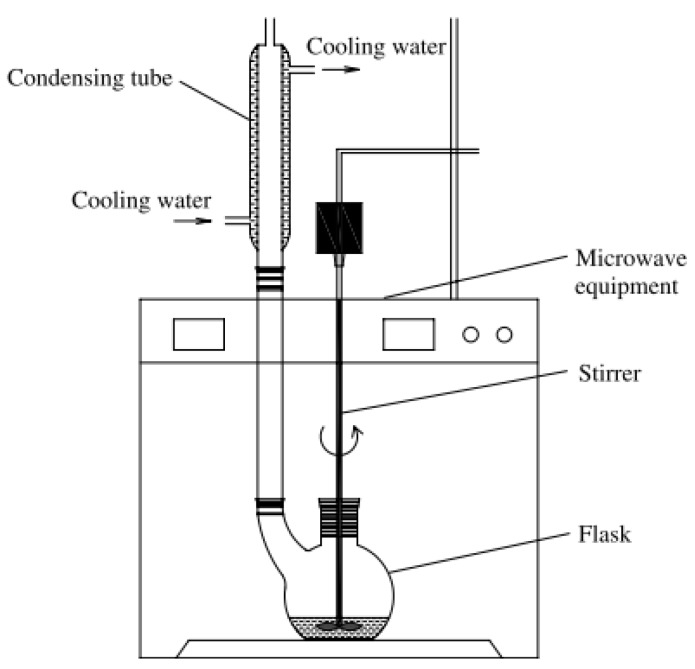
Schematic diagram of the microwave equipment.

Polyphenol compounds were extracted from fruit hull of *C. oleifera*, using a MAS-II microwave extraction system (Sineo Microwave Equipment Co., Ltd., Shanghai, China) at a frequency of 2,450 MHz. Its schematic diagram is shown in Figure 3. The extraction variables evaluated were extraction temperature, extraction time and liquid:solid ratio. Five hundred mL flasks were used to extract 10 g of ground fruit hull. Water was used as a solvent. The crude fruit hull extracts were allowed to cool to room temperature before centrifugation (Model LD5-2B, Jingli equipment Co., Ltd., Beijing, China) at 4,800 rpm for 30 min. The supernatant was collected for polyphenol analysis.

### 3.3. Polyphenol Determination

Polyphenols content was determined according to the method of Jayaprakasha *et al.* [19]. Aliquots (0.5 mL) of 10-fold-diluted extracts were mixed with 10-fold-diluted Folin-Ciocalteu reagent (2.5 mL) and 7.5% sodium carbonate (2 mL). The mixture was allowed to stand for 30 min at room temperature before the absorbance was measured spectrophotometrically at 760 nm. A mixture of water and reagents was used as a blank. The polyphenol content was expressed as mg of gallic acid equivalents (GAE)/g of dry material.

### 3.4. HPLC Analysis of Extracts

The contents of phenolic compounds in the extracts of *C. oleifera* fruit hull were determined by HPLC, performed with a Shimadzu LC-20AB instrument equipped with a binary pump (Shimadzu, Japan). The analyses were carried out on a Hypersil ODS column (5 μm, 250 mm × 4.6 mm) column. Extracts were filtered through a 0.45 µm filter before use. The mobile phase was composed of solvent (A) water (0.1% TFA, v/v) and solvent (B) CH_3_CN. The gradient condition was: 0-5th minutes 5% B, 5th–10th min 5%–10% B. Other chromatographic conditions were as follows: flow rate at 1 mL/min; volume injected 20 µL; room temperature; detection at 280 nm. Retention times and spectra were compared with those of pure standards. Each sample analysis was repeated three times.

### 3.5. Total Flavonoid Content

Total flavonoid content was determined according to a known method [20] with a slight modification; aliquots of 10-fold-diluted extracts (2 mL) were mixed with 5% NaNO_2_ (0.3 mL). After 6 min at 25 °C, Al(NO_3_)_3_ (0.3 mL, 10%) was added. The reaction mixture was treated with 1M NaOH (4 mL) for 6 min, then diluted to 10 mL with 50% ethanol solution, and the absorbance was read at 500 nm. Quercetin was used as the standard and the total flavonoid content was expressed as quercetin equivalents (QE) mg/g dry material.

### 3.6. Statistical Analysis

RSM was performed using the Design-Expert software (Version 7.1.3, Stat-Ease) program. A central composite design (CCD) was used to investigate the effects of three independent variables (extraction temperature, extraction time and liquid:solid ratio) at five levels on the dependent variables (polyphenol yield). A CCD uses the method of least squares regression to fit the data to a quadratic model. The quadratic model for each response was as follows:

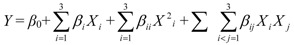
(2)
where *Y* is the predicted response, *β*_0_ a constant, *β_i_* the linear coefficient, *β_ii_* the quadratic coefficient, *β_ij_* the interaction coefficient of variables *i* and *j*, and *X_i_* and *X_j_* are independent variables (*i* ≠ *i*). The software uses this quadratic model to build response surfaces. The adequacy of the model was determined by evaluating the lack of fit, coefficient of determination (*R*^2^) and the Fisher test value (*F*-value) obtained from the analysis of variance (ANOVA) generated by the software. Statistical significance of the model and model parameters was determined at the 5% probability level (*a* = 0.05). Three-dimensional response surface plots and contour plots were generated by keeping one response variable at its optimal level and plotting that against two factors (independent variables). The codes used in the response surface analysis and the corresponding parameter values are given in Table 4. The complete design consisted of 20 experimental points, including six replicates of the centre point.

**Table 4:** Independent variable values of the process and their corresponding levels.

Independent variable	Symbol				Levels				
	Uncoded	Coded			−1.68	−1	0	1	1.68
Extraction time (min)	*X*_1_	*x*_1_			8.18	15	25	35	41.82
Liquid:solid ratio (v/w)	*X*_2_	*x*_2_			11.59	15	20	25	28.41
Extraction temperature (°C)	*X*_3_	*x*_3_			53.18	60	70	80	86.82

## 4. Conclusions

An efficient microwave-assisted extraction process has been developed for the extraction of polyphenols from *C. oleifera*. CCD was successfully employed to optimize the extraction parameters. The best conditions were shown to be liquid:solid ratio of 15.33:1 (mL/g), microwave extraction time of 35 min, and microwave extraction temperature of 76 °C. The maximum polyphenol yield was 15.05 ± 0.04% (*n* = 5) under these optimal conditions. This study can be useful for the development of industrial extraction of polyphenols from *C. oleifera*, including further studies concerning the optimal number of sequential steps to enhance the efficacy of a potential large-scale extraction system.
